# Tanshinone IIA inhibits proliferation and migration by downregulation of the PI3K/Akt pathway in small cell lung cancer cells

**DOI:** 10.1186/s12906-024-04363-y

**Published:** 2024-01-31

**Authors:** Yuxin Jiang, Yanli Bi, Lingjie Zhou, Senwen Zheng, Tingting Jian, Jian Chen

**Affiliations:** 1 School of Basic Medical Sciences and Forensic Medicine, Hangzhou Medical College, No. 481 Binwen Road, Binjiang District, Hangzhou, 310053 Zhejiang China; 2 Department of Clinical Laboratorial Examination, Air Force Hangzhou Special Service Recuperation Center Sanatorium Area 3, Hangzhou, Zhejiang China

**Keywords:** Tanshinone IIA, Small cell lung cancer, Metastasis, Epithelial-to-mesenchymal transition, PI3K, Akt

## Abstract

**Background:**

Small cell lung cancer (SCLC) is the most malignant lung cancer type. Due to the high rates of metastasis and drug resistance, effective therapeutic strategies remain lacking. Tanshinone IIA (Tan IIA) has been reported to exhibit anti-tumor activity. Therefore, this study investigated the ability and underlying mechanism of Tan IIA to inhibit the metastasis and proliferation of SCLC.

**Methods:**

H1688 and H446 cells were treated in vitro with Tan IIA (0, 1, 2 and 4 µM) or LY294002 (10 µM) for 24, 48, 72 h. H1688 and H446 cell migration was evaluated in wound healing and transwell migration assays. RNA-sequencing helped assess gene expression. BALB/c nude mice were injected with H1688 cells and treated with the Tan IIA group (10 mg/kg/day) or a control. Expression of E-cadherin, vimentin and PI3K/Akt signaling pathway proteins in tumors and H1688 was investigated by immunohistochemical analysis and western blot.

**Results:**

Tan IIA inhibited H1688 and H446 cell proliferation without inducing apoptosis and suppressed H1688 and H446 cell migration. E-cadherin expression was increased, while vimentin expression was reduced after administration of Tan IIA. RNA-sequencing revealed that some genes related with the PI3K/Akt signaling pathway were altered using Tan IIA treatment. Furthermore, western blot helped detect PI3K and p-Akt expression was also reduced by Tan IIA treatment. Tan IIA inhibited tumor growth in vivo. Moreover, Tan IIA increased tumoral expression of E-cadherin accompanied by PI3K and p-Akt downregulation.

**Conclusion:**

Tan IIA suppresses SCLC proliferation and metastasis by inhibiting the PI3K/Akt signaling pathway, thereby highlighting the potential of Tan IIA as a new and relatively safe drug candidate to treat SCLC.

**Supplementary Information:**

The online version contains supplementary material available at 10.1186/s12906-024-04363-y.

## Background

As one of the most common types of malignant tumors in clinical practice, lung cancer incidence and mortality rate in China has been increasing in recent years due to lifestyle changes and the severity of environmental pollution [1]. Although small cell lung cancer (SCLC) accounts for only about 20% of lung cancers, it is the most malignant and deadly form, with less than 6% 5-year survival rate [2]. The current standard first-line treatment combines etoposide and platinum-based chemotherapy regimen [3]. However, this treatment remains limited for SCLC since most patients develop recurrent disease accompanied by tumor metastasis. In addition, these anti-tumor drugs also show toxicity against normal cells with drug resistance to additional therapies [4]. Therefore, novel therapeutic drugs are urgently required to improve the efficiency of SCLC therapy.

Chinese herbal medicines and their extracts have been widely utilized to treat multiple tumors in clinical practice due to their low toxicity and good efficacy [5]. Previous studies have indicated that Chinese herbs can improve the overall survival (OS) and quality of life of SCLC patients [6, 7]. Thus, using Chinese herb-derived active compounds to treat SCLC is a promising approach. Multiple compounds extracted from Chinese herbs have anti-tumor abilities, including Tanshinone IIA (Tan IIA) [7–10]. However, since there are hundreds of components in Chinese herbal medicines, it is challenging to elucidate the underlying molecular mechanism, which is why TCM is not widely accepted in Western countries.

Tan IIA is one of the main active components of *Salvia miltiorrhiza*. Tan IIA has a variety of pharmacological activities, such as anti-inflammation, anti-oxidation, and neuroprotection [11, 12]. Tan IIA also exhibits anti-tumor activity in various cancers, including non-small cell lung cancer (NSCLC) [10]. However, its anti-tumor effect on SCLC has not been reported.

The highly metastatic nature of SCLC is a major cause of the high mortality in SCLC patients [13]. However, the molecular mechanisms underlying this characteristic still need to be better understood. Previous studies reveal multiple signaling pathways in tumor metastasis are associated with epithelial-to-mesenchymal transition (EMT) [14]. Based on a recent study, the PI3K/Akt pathway is critical in bioenergetic processes and metastasis of SCLC cells [15]. Moreover, the PI3K/Akt signaling blockade could inhibit tumor proliferation and promote chemosensitization of SCLC [16]. Therefore, PI3K/Akt signaling represents a novel therapeutic target in SCLC. One study demonstrated that Tan IIA treatment considerably suppressed the proliferation and migration of cholangiocarcinoma cells by inhibiting the PI3K/Akt pathway [17]. Other reports indicated that Tan IIA activates the PI3K/Akt pathway [18, 19].

Therefore, this study investigated that Tan IIA exerts an anti-tumor effect in small cell lung cancer cells from human and mouse tumor xenograft model by activating the PI3K/Akt pathway.

## Methods

### Chemicals and reagents

Tanshinone IIA (Tan IIA) was obtained from the Zhejiang Institute for Drug Control (Hangzhou, Zhejiang, China). The PI3K inhibitor LY294002 was purchased from Selleck (Houston, Texas, USA). Roswell Park Memorial Institute (RPMI) 1640 medium and fetal bovine serum (FBS) was obtained from GIBCO (Carlsbad, CA, USA). TRIzol reagent was obtained from Invitrogen. 3-(4,5-Dimethylthiazol-2-yl)-2,5-diphenyltetrazolium bromide (MTT) and dimethyl sulfoxide (DMSO) were purchased from Sigma (St. Louis, MO, USA). We used primary antibodies against the following proteins: E-cadherin, vimentin (Abcam, Waltham, MA, USA); Akt and p-Akt (Ser473) (Cell Signaling Technology, Danfoss, MA, USA); PI3K and GAPDH (Abcam). Secondary anti-mouse and anti-rabbit antibodies were provided by Pierce (Carlsbad, CA, USA).

### Cell lines and culture conditions

A human small cell lung cancer (SCLC) cell lines (H1688 and H446), a human normal lung epithelial cell line (BEAS-2B), and a human normal bronchial epithelial cell line (HBE) were obtained from Zhejiang University, School of Medicine (Hangzhou, Zhejiang, China). The four cell lines were cultured in RPMI-1640 supplemented using 10% FBS, 100 U/mL streptomycin and 100 µg/mL penicillin at 37 °C under 5% CO_2_ in a humidified incubator based on our previous study [20].

### Cell viability assay

Cells were seeded in 96-well plates (1 × 10^4^ cells/well), incubated at 37 °C for 24 h, and then treated with various doses of Tan IIA (0, 1, 2, 4 and 6 µmol/L) for various time points (24, 48 and 72 h) or LY294002 (10 µmol/L) for 48 h. After treatment, 10 µl MTT (5 mg/ml) was added to each well and the cells were incubated for another 4 h at 37 °C. Then, the culture medium in each well was replaced with 200 µl DMSO and the optical density (OD) value was measured at 570 nm. Cell viability rate (%) = OD_test group_/OD_control group_×100%.

### Colony formation assay

H1688 and H446 cells were plated onto 6-well plates (5 × 10^2^ cells/well) and treated with Tan IIA (0, 1, 2, 4 and 6 µmol/L) or LY294002 (10 µmol/L) for 48 h. Then, the cells were cultured in a complete medium for 14 days, with medium renewal every three days. The cells were fixed with 4% paraformaldehyde and stained with 0.5% crystal violet to count the number of clones through direct visual inspection.

### Flow cytometric analysis of cell apoptosis

Apoptotic cells were detected using an Annexin V-FITC/propidium iodide (PI) staining kit (Yeasen Biotech Co., Ltd.). H1688 and H446 cells were seeded in 6-well plates (5 × 10^5^ cells/well) and cultured for 24 h before Tan IIA (0, 1, 2, 4 and 6 µmol/L) or LY294002 (10 µmol/L) treatment. After 48 h, cells were released from the plate through trypsin digestion without EDTA, harvested, and washed twice using cold PBS. Subsequently, the cells were stained with 5 µL Annexin V and 10 µL PI in 500 µL binding buffer for 15 min at room temperature in the dark, and analyzed by using flow cytometry. Apoptosis rate (%) = (early apoptotic cells + late apoptotic cells)/total number of cells × 100%.

### Wound healing assay

H1688 and H446 cells were seeded in 6-well plates (5 × 10^5^ cells/well) and cultured to 100% confluence. Then, the cell monolayer was scratched with a 200-µL pipette tip to generate a wound. The wounded monolayer was washed twice using PBS and incubated with Tan IIA (0, 1, 2 and 4 μmol/L) or LY294002 (10 µmol/L). The wound was photographed at the same location using an inverted microscope at 0 and 48 h, and the width of the open wound in three fields was measured. The migration index (%) = (‘0 h’ wound width − ‘48 h’ wound width)/‘0 h’ wound width × 100%.

### Transwell migration assay

H1688 and H446 cells were suspended in a serum-free medium containing the indicated concentration of Tan IIA (0, 1, 2 and 4 µmol/L), or LY294002 (10 µmol/L) in upper chambers. In contrast, 600 µL medium with 10% FBS was added to the lower chambers. After incubation for 48 h, cells in the upper chamber were carefully removed, while cells on the lower chamber surface were fixed with 4% paraformaldehyde and stained with 0.5% crystal violet. The migrated cells were photographed and counted in three random fields under a light microscope.

### Western blot analysis

H1688 and H446 cells were washed twice with PBS and lysed in radioimmunoprecipitation (RIPA) buffer to extract the total proteins. The protein content was measured with a Bradford protein assay kit. Equal proteins amount were separated by sodium dodecyl sulfate polyacrylamide gel electrophoresis (SDS-PAGE) and transferred to a polyvinylidene fluoride (PVDF) membrane for incubating with diluted primary antibodies (1:1000) at 4℃ overnight. Then, the membrane was twashed three times with TBST at room temperature and incubated with the appropriate horseradish peroxidase (HRP)-linked secondary antibody for 1 h at room temperature. Proteins were visualized using enhanced chemiluminescence detection reagents (Biosharp, Guangzhou, China).

### RNA extraction and RNA-sequencing (RNA-seq)

H1688 cells were incubated in 10-cm culture dishes (2 × 10^6^ cells/dish) for 24 h before Tan IIA (0 and 2 µmol/L) treatment for 48 h. After washing with PBS, the cells were lysed in 1 mL Trizol solution to extract the total RNA.

An RNA-seq transcriptome library was prepared with 1 µg total RNA in a TruSeqTM RNA sample preparation kit (Illumina, CA, USA) according to the manufacturer’s instructions. After verifying the integrity and purity of RNA, the mRNA was randomly fractured into approximately 200-bp fragments and transcribed into double-stranded DNA using added adapters. The resulting DNA products were sequenced with an Illumina HiSeq 4000 by Majorbio Ltd. The transcripts per million reads (TPM) method helped determine the expression of each transcript. Genes with |log2(fold change, FC) | values > 1 were identified as significantly differentially expressed genes (DEGs). Gene ontology analysis and Kyoto Encyclopedia of Genes and Genomes (KEGG) pathway enrichment analysis were performed with the Database for Annotation, Visualization, and Integrated Discovery (DAVID) described previously [21].

### In vivo tumorigenic assay and immunohistochemistry

Animal experiments were conducted by the guidelines for Care and Use of Laboratory Animals approved by the Hangzhou Medical College Ethics Committee.

Ten Male BALB/nude mice (aged five weeks) were purchased from Shanghai Laboratory Animal Center (Shanghai, China) and housed in a dedicated SPF facility under alternating 12 h periods of light and darkness at a constant temperature of 21 ± 2 °C with 45 ± 10% humidity. H1688 cells (1 × 10^7^) in 200 µL of PBS were injected subcutaneously into the right axillary region. One week after cell implantation, the nude mice were randomly divided into Tan IIA and control groups (*n* = 5 mouse/group). Tan IIA group received an intraperitoneal injection of 10 mg/kg Tan IIA every other day (11 in total). The control group received the same volume of saline. The sample size calculation was dependent on our previous study with five rats per group deemed necessary [20].

Mice weights and tumor volumes were measured every other day. Tumor sizes were calculated using the following equation: a × b^2^/2, where a and b represent the long and short diameters of the tumor, respectively. After treatment, the mice were humanely sacrificed by cervical dislocation under anesthesia (1% pentobarbital sodium salt, 30 mg/kg). This process involved three investigators: The first investigator divided the mice following the randomization table. Another researcher performed the anesthetic procedure, while a third investigator was responsible for the injection procedure.

Tumor specimens (three from each group) were collected, weighed, fixed in 4% paraformaldehyde for 24 h at room temperature, and embedded within paraffin. The paraffin-embedded tissues (three µm) were subjected to immunohistochemistry analyses with antibodies against E-cadherin, vimentin, PI3K and p-Akt using a 3, 3’-diaminobenzidine substrate kit (Solarbio, Beijing, China) following the manufacturer’s protocol. Slides were photographed under a light microscope (Nikon Corporation, Japan). The Image-Pro Plus 6.0 software helped the densitometry immunohistochemical staining. Mean optical density OD = total OD/area of area of interest (AOI).

### Statistical analysis

Data were presented as mean ± standard deviation (SD) of least three independent experiments. Statistical analysis was performed with GraphPad Prism 6. A two-tailed unpaired Student’s t-test helped compare the two groups. A one-way ANOVA test was used to differentiate the multiple groups, followed by the *post-hoc* Tukey test. *P* < 0.05 was considered a statistically significant difference.

## Results

### Tan IIA inhibited the growth of H1688 and H446 cells

**Fig. 1:**
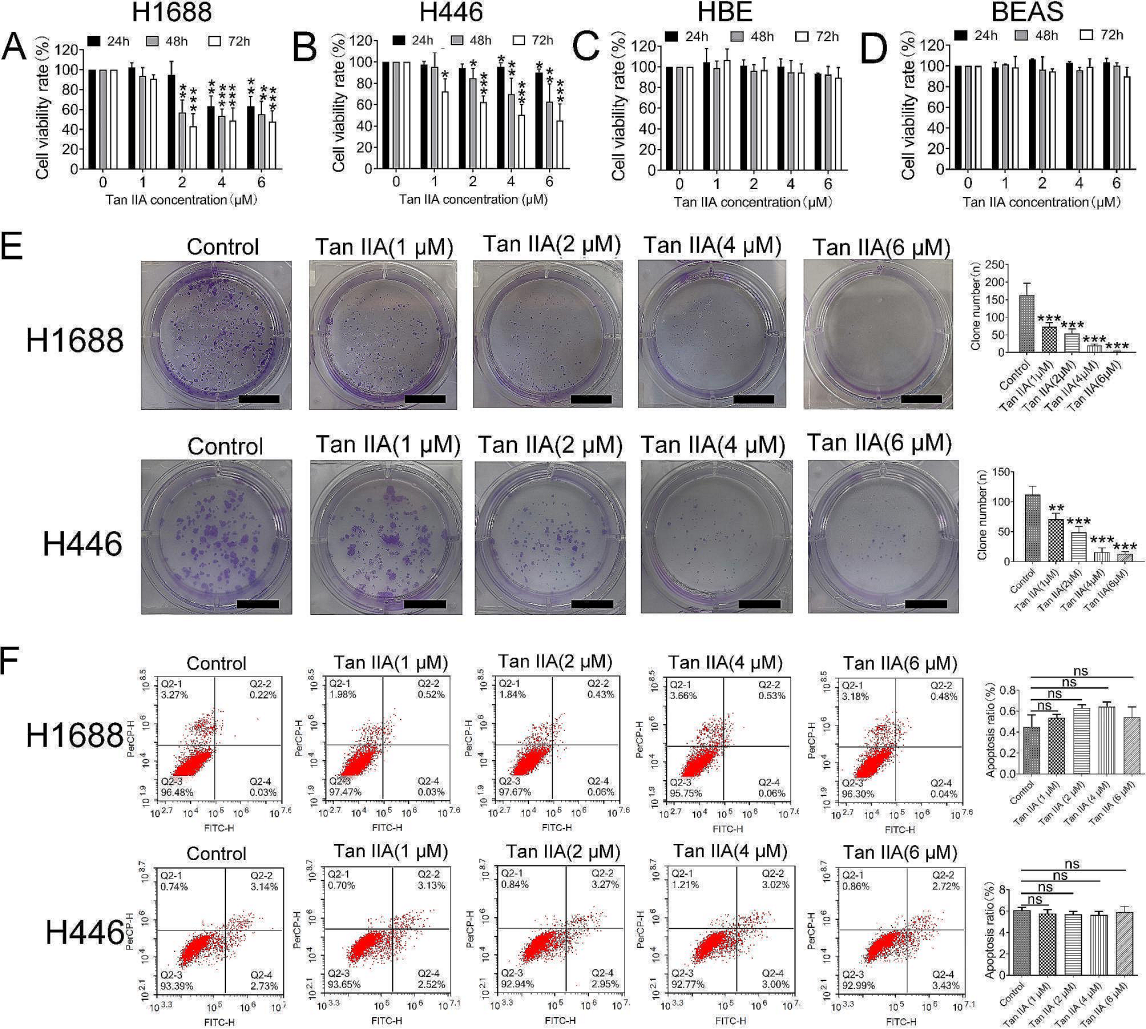
Tan IIA inhibited H1688 and H466 cell proliferation. H1688, H446, BEAS-2B and HBE cells treated with Tan IIA (0, 1, 2, 4 or 6 µM) for 24, 48 or 72 h. (**A-D**) H1688, H446, BEAS-2B and HBE cell viability determined by MTT assay (*n* = 3). (**E**) Colony formation assays of H1688 and H446 cell growth; scale bars: 10 mm, (*n* = 3). (**F**) Flow cytometric analysis of apoptotic cells double-stained with Annexin V-FITC and PI. Compared with the control group (*n* = 3), ns > 0.05, * *P* < 0.05, ** *P* < 0.01, *** *P* < 0.001

MTT assay was used to detect the viability of multiple cells after treatment with various concentrations of Tan IIA for 24, 48, and 72 h. As shown in Fig. 1A, Tan IIA inhibited H1688 cell growth in a dose- and time-dependent manner. A high Tan IIA (4 and 6 µM) dosage induced approximately 50% more growth inhibition after 24, 48, and 72 h of treatment than the control group (*P* < 0.01 and *P* < 0.001). In addition, Tan IIA (2 µM) also significantly suppressed H1688 and H446 cell growth after 72 h of treatment compared with the control group (Fig. 1A and B; *P* < 0.001). The growth ability of H1688 and H446 cell was also impaired after Tan IIA treatment (Fig. 1E; *P* < 0.01 and *P* < 0.001). However, Tan IIA treatment for 48 h did not affect on apoptosis induction detected by flow cytometry (Fig. 1F; *P* > 0.05). In contrast, no marked cytotoxic effects were observed in HBE and BEAS cell lines exposed to the same Tan IIA concentrations (1, 2, and 4 µM) (Fig. 1C and D; *P* > 0.05). Therefore, low Tan IIA (1, 2 and 4 µM) concentrations were utilized in subsequent experiments.

### Tan IIA inhibited H1688 and H446 cell migration and reduced EMT

**Fig. 2:**
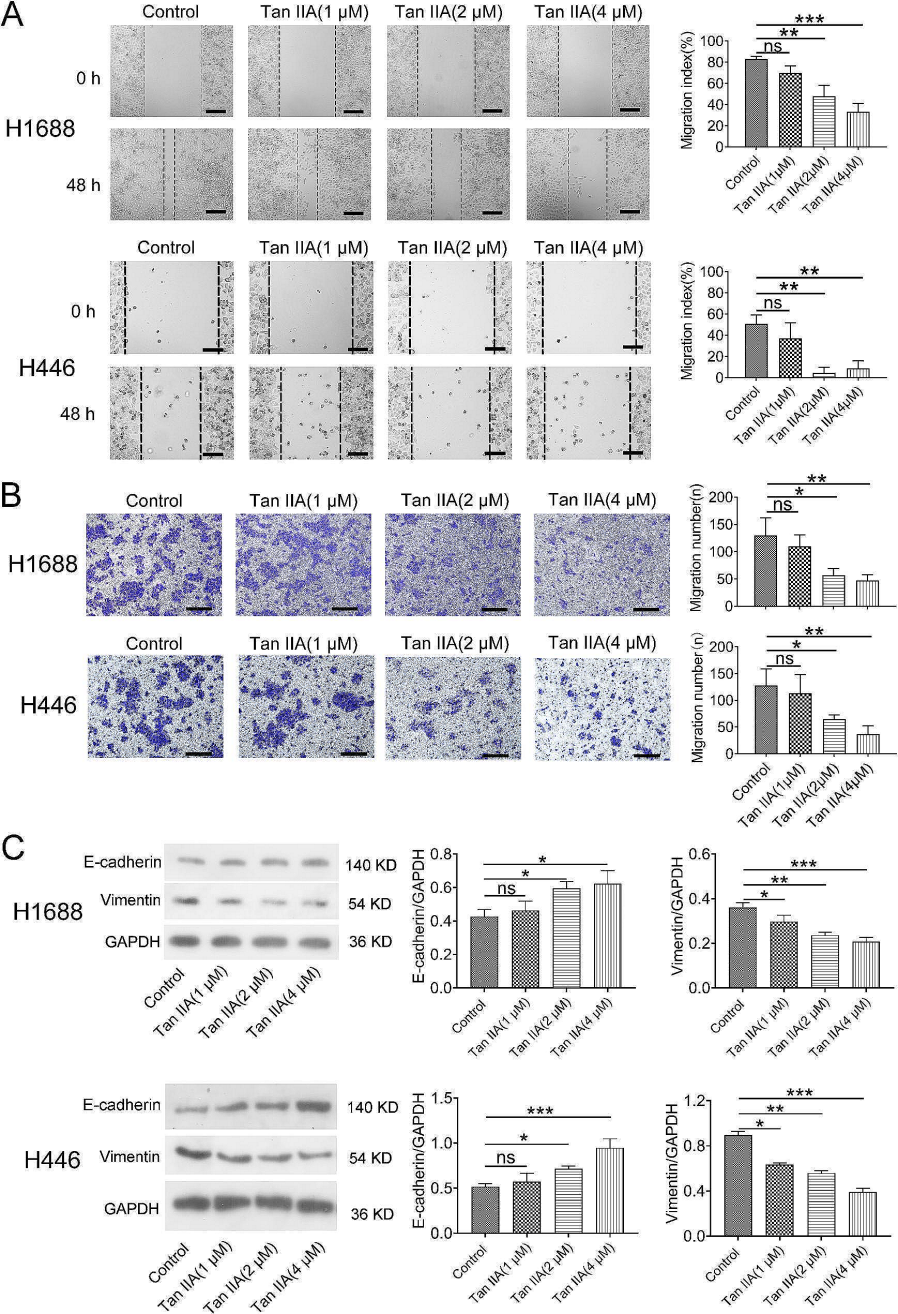
The migration of H1688 and H446 cells was reduced by Tan IIA treatment by suppression of the EMT process. (**A**) Migration of H1688 and H446 cells treated with Tan IIA (0, 1, 2 and 4 µmol/L) determined by wound healing assay. Representative sections indicate a significant decrease in migration index after 48 h of Tan IIA treatment (left); scale bars = 100 μm. Statistical analysis (*n* = 3; right). (**B**). Migration of H1688 and H446 cells treated with Tan IIA (0, 1, 2 and 4 µmol/L) for 48 h determined by Transwell assays. Cells that migrated across the Transwell membrane were stained with crystal violet, which showed a marked decrease after 48 h of Tan IIA (2 and 4 µmol/L) treatment (left); scale bars: 100 μm. Statistical analysis (*n* = 3; right). (**C**). E-cadherin expression was significantly increased after 48 h of Tan IIA (2 and 4 µmol/L) treatment, whereas the expression of vimentin was decreased in the Tan IIA-treated groups when compared with the control group; ns > 0.05, * *P* < 0.05, ** *P* < 0.01, *** *P* < 0.001

Migration is one of the most essential characteristics of EMT. Wound healing assays revealed that Tan IIA (2 and 4 µmol/L) treatment for 48 h significantly reduced cell migration when compared with the control group (Fig. 2A; *P* < 0.01 and *P* < 0.001). Furthermore, migrated cells were significantly reduced after Tan IIA treatment (2 and 4 µmol/L) for 48 h (Fig. 2B; *P* < 0.05 and *P* < 0.01). These findings depicted that Tan IIA inhibits H1688 and H446 cancer cell migration. Since E-cadherin and vimentin are important in the EMT process, the expression of these proteins was measured. Figure 2C revealed that the expression levels of vimentin were decreased in cells treated with Tan IIA (*P* < 0.01 and *P* < 0.001). In contrast, E-cadherin expression was more elevated than the control group (*P* < 0.05 and *P* < 0.001).

### Influence of Tan IIA on the transcriptome of H1688 cells

**Fig. 3:**
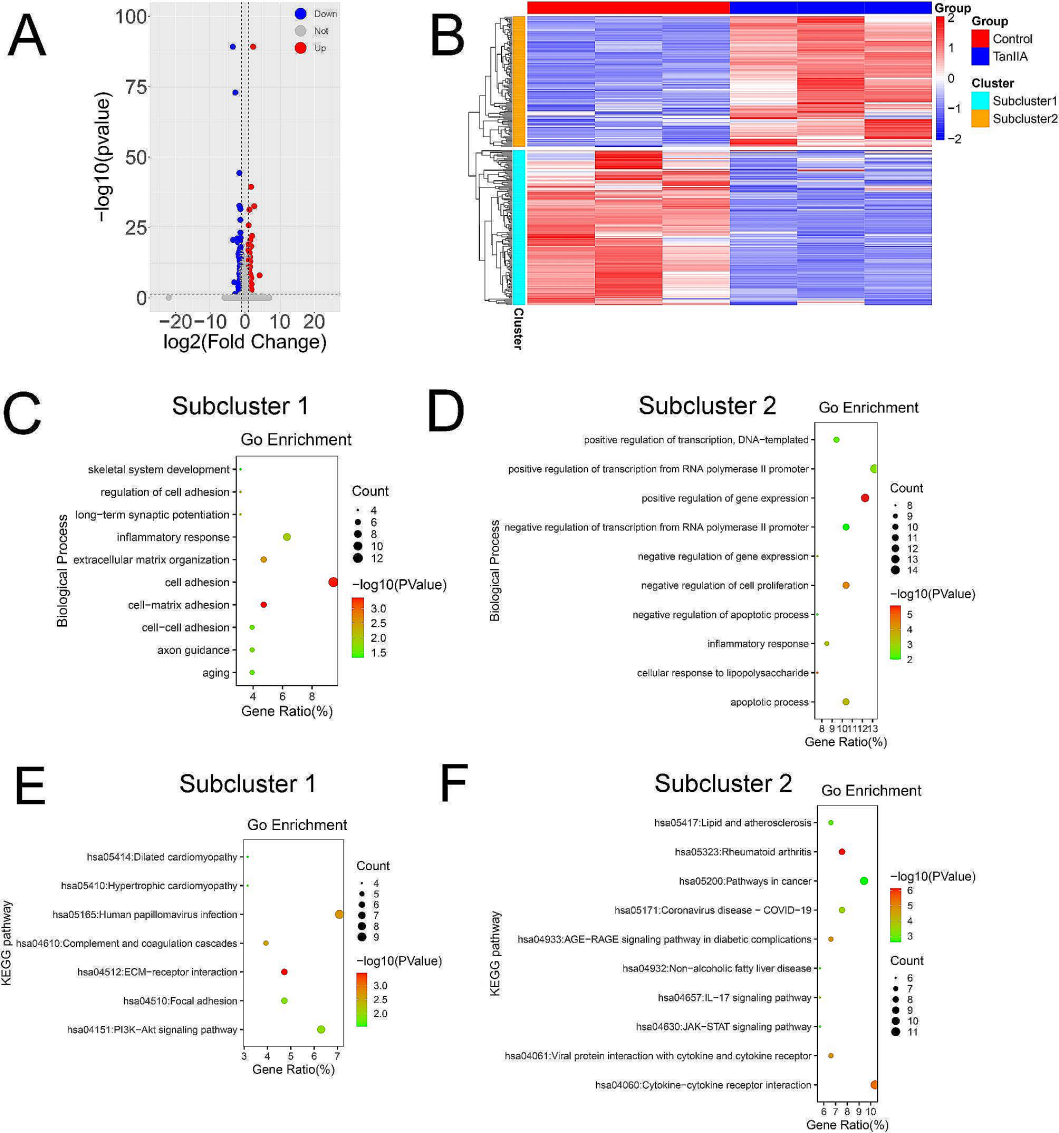
Influence of Tan IIA on the transcriptome of H1688 cells. (**A**) Volcano plots of differentially expressed genes (DEGs) in the Tan IIA group (*n* = 3) compared with the control group (*n* = 3). (**B**) Subcluster analysis of the DEGs shown as a heatmap. (**C, D**) Biological process (BP) enrichment analyses of representative DEGs in subcluster 1 and 2. (**E, F**) Kyoto Encyclopedia of Genes and Genomes (KEGG) pathway enrichment analyses of representative DEGs in subcluster 1 and 2

To identify the genes with expression altered by Tan IIA treatment of H1688 cells, samples from the control group (*n* = 3) and Tan IIA group (*n* = 3) were used for RNA-seq analysis. In total, 256 DEGs were detected by comparison of the Tan IIA and control groups (Fig. 3A). These genes were divided into subcluster 1, with 139 downregulated genes and subcluster 2, with 117 upregulated genes in Tan IIA group (Fig. 3B). BP analysis revealed that the DEGs in subcluster 1 were mainly enriched in cell adhesion, while those in subcluster 2 were mainly enriched in controlling RNA transcription and gene expression (Fig. 3C and D). Furthermore, KEGG pathway analysis revealed that DEGs in subcluster 1 participated in cell adhesion and EMT, such as the PI3K/Akt pathway (Fig. 3E). Although the DEGs in subcluster 2 were enriched in some diverse signaling pathways which were not closely related to cell adhesion and proliferation (Fig. 3F). The DEGs in subcluster 1 were selected for addition analysis.

### Tan IIA regulates SCLC progression via the PI3K/Akt pathway

**Fig. 4:**
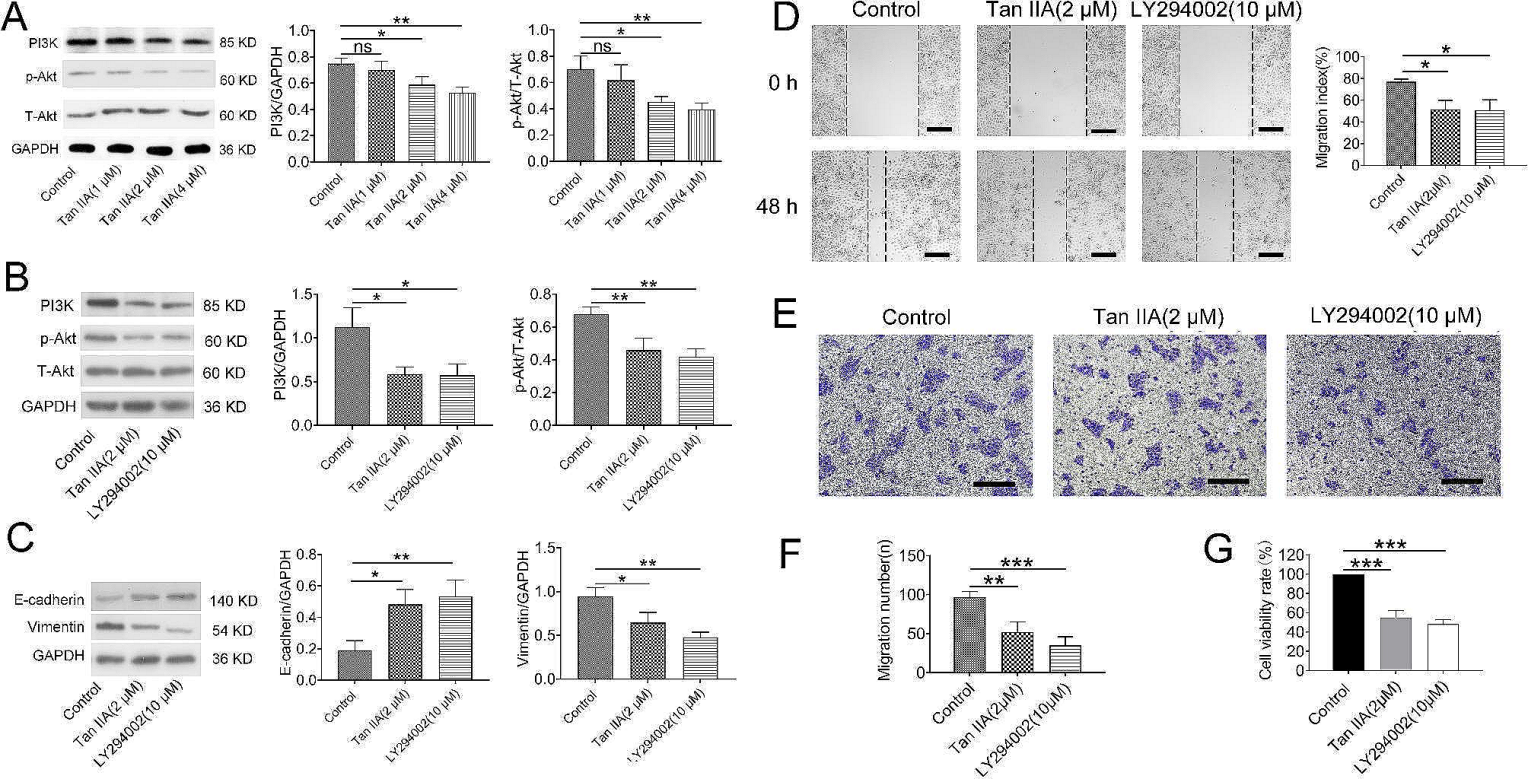
Tan IIA inhibited cancer cell proliferation and migration via the PI3K/Akt signaling pathway. (**A**) The protein expression levels of PI3K, Akt and p-Akt were measured after Tan IIA treatment (0, 1, 2 and 4 µM). (**B, C**) The expression of PI3K/Akt-related proteins, E-cadherin and vimentin was detected in H1688 cells after exposure to Tan IIA (2 µM) or LY294002 (10 µM) for 48 h. The effect of LY294002 on cell migration (**D, E, F**) and viability (**G**) were analyzed. Compared with control (*n* = 3), ns > 0.05, * *P* < 0.05, ** *P* < 0.01, *** *P* < 0.001

To explore the molecular mechanism by which Tan IIA regulates SCLC progression, we first investigated the Tan IIA effect on the PI3K/Akt pathway in H1688 cells. The results depicted that Tan IIA (2 and 4 µmol/L) treatment reduced PI3K and p-Akt/Akt levels in H1688 cells (Fig. 4A; *P* < 0.05 and *P* < 0.01). Then, we used the specific inhibitor LY294002 to investigate the potential association of the PI3K/Akt pathway with SCLC progression. Tan IIA and LY294002 blocked the PI3K/Akt pathway and suppressed EMT by regulating the expression of E-cadherin and vimentin (Fig. 4B and C; *P* < 0.05 and *P* < 0.01). Tan IIA and LY294002 also significantly reduced cell proliferation and repressed H1688 cell migration (Fig. 4D and G; *P* < 0.05, *P* < 0.01 and *P* < 0.001). Thus, Tan IIA suppressed cell proliferation and migration by blocking the PI3K/Akt pathway.

### Tan IIA inhibited xenograft implantation tumors and EMT in vivo

**Fig. 5:**
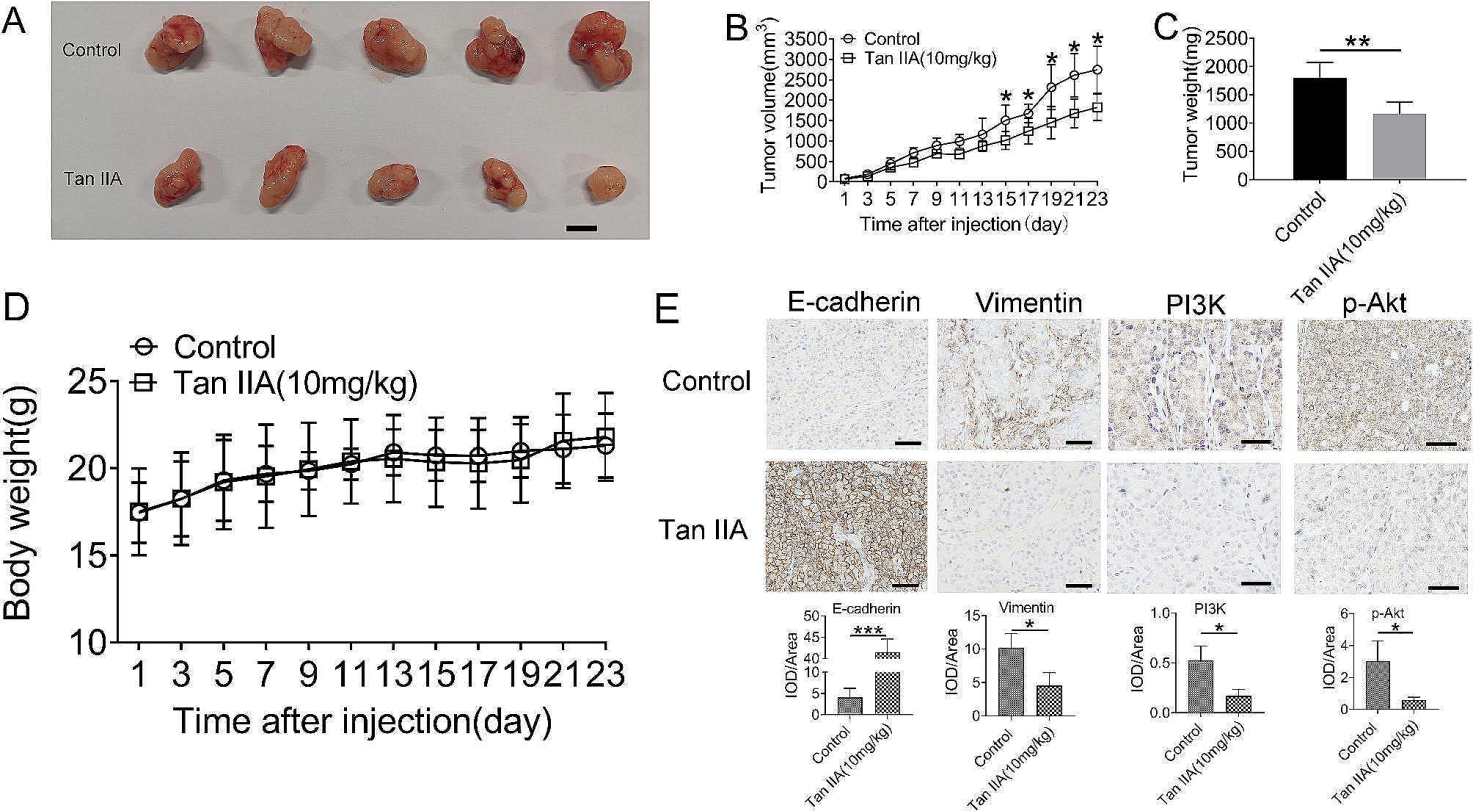
Tan IIA attenuated tumor growth with a high level of safety in vivo. (**A**) Representative images of tumor from the control group and the Tan IIA (10 mg/kg) group; scale bars: 1 cm, (*n* = 5). (**B**) The tumor width and length were measured every other day (*n* = 5). (**C**) The subcutaneous tumors were weighed immediately at the end of the study. (**D**) Body weight was not obviously altered after treatment with Tan IIA (*n* = 5). (**E**) Immunohistochemical analysis of the expression of E-cadherin, vimentin, PI3K and p-Akt; scale bar: 50 μm. Compared with control (*n* = 3), * *P* < 0.05, ** *P* < 0.01, *** *P* < 0.001

To evaluate the antitumor effect of Tan IIA in vivo, we used an H1688 tumor-bearing xenograft model in mice. As shown in Fig. 5A-C, the tumor volume and weight in the Tan IIA-treated mice were reduced compared with the control group (*P* < 0.05 and *P* < 0.01). Additionally, the toxicity of Tan IIA was assessed by monitoring body weight and serum biochemical parameters between the two groups. Compared with the control group, there was no noticeable decrease in body weight after Tan IIA treatment (Fig. 5D; *P* > 0.05). Moreover, the two groups had no significant difference in serum biochemical parameters (Table 1). Moreover, immunohistochemistry analysis revealed that E-cadherin expression was remarkably elevated. Furthermore, vimentin, PI3K and p-Akt expression was also suppressed in tumor tissue treated with Tan IIA (Fig. 5E; *P* < 0.05 and *P* < 0.001). Thus, these in vivo findings were consistent with the in vitro data.

**Table 1:** Effects of Tan IIA treatment on the serum levels of biochemical parameters in the tumor xenograft model (*n* = 5)

Groups	CREA (µmol/L)	UREA (mmol/L)	ALT (U/L)	AST (U/L)	ALP (U/L)	LDH (U/L)
Control	48.6 ± 9.21	10.39 ± 2.51	28.20 ± 9.65	162.80 ± 40.83	83.40 ± 10.69	3262.00 ± 320.92
Tan IIA(10 mg/kg)	52.8 ± 6.91	10.83 ± 1.27	31.60 ± 12.66	145.60 ± 22.47	64.80 ± 5.07	2837.40 ± 399.20

Data are presented as the mean ± SD (*n* = 5). ALT: alanine aminotransferase; AST: aspartate aminotransferase; ALP: alkaline phosphatase; CREA: creatinine; LDH: lactate dehydrogenase

## Discussion

SCLC is an aggressive cancer of neuroendocrine origin that progresses rapidly, often with endocrine abnormalities or carcinoid syndrome. Approximately two-thirds of patients have metastases by diagnosis [22]. Lung cancer metastasis is the process by which a primary malignant tumor in the lung is seeded far away from its primary site through multiple pathways [13]. Despite a high response rate to the initial chemotherapy and radiotherapy, most patients present with advanced disease and rapidly develop resistance to distant metastases [17, 23]. Therefore, new effective drugs for treating of SCLC with low toxicity are urgently needed. In the present study, Tan IIA inhibited the migration and proliferation of SCLC, providing a new avenue for developing SCLC treatment.

Tan IIA is the main active component derived from *Salvia miltiorrhiza*. Previous studies have indicated that Tan IIA exerts anti-tumor effects [24] and inhibits the growth of various tumor cell lines, such as liver, pancreatic and colorectal cancer [12]. However, the effects of Tan IIA on SCLC and the potential anti-metastatic mechanisms are unknown. One study suggests EMT is associated with the metastatic process of NSCLC and SCLC [25]. During EMT, epithelial cells shed their connections to neighboring cells, changing from apical-basal polarity to anterior-posterior polarity, and adapting the properties of migrating mesenchymal cells. Adhesion is promoted in cells undergoing EMT by epithelial genes repression, leading to their separation from adjacent cells. The classical epithelial marker E-cadherin is a critical component of adherent junctions and is the most prominent EMT inhibition target [26]. E-cadherin can affect the strength of cellular adhesion and promote cellular motility [27]. In contrast, vimentin, an abundant cytoplasmic intermediate filament protein, is related to cancer metastasis by stabilizing cell connections [28]. Our study demonstrated that Tan IIA inhibited tumor growth and suppressed cell migration by increasing E-cadherin expression and decreasing vimentin expression. We showed that Tan IIA significantly reduced the cell migration in wound healing and Transwell migration assays. Furthermore, the same Tan IIA concentrations did not affect the ability of normal lung cell lines. Thus, Tan IIA has relatively low toxicity.

In addition, BP analysis revealed that the DEGs downregulated by Tan IIA are enriched in the cell adhesion and inflammation categories associated with EMT. Furthermore, KEGG pathway enrichment analysis indicated that these DEGs are closely related to the PI3K/Akt pathway. PI3K is a major intracellular signaling pathway responsible for various vital cellular processes aberrantly activated in cancer while contributing to tumorigenesis and progression [29]. The PI3K/Akt pathway can be aberrantly activated through multiple mechanisms. Once activated, Akt phosphorylates many substrates, including the mammalian target of rapamycin (mTOR). This downstream Akt effector activates many proteins and promotes cancer progression [30]. Some herbal medicines can inhibit tumors by modulating the PI3K/Akt signaling pathway. For example, Shikonin inhibits the migration and invasion of glioblastoma cells by targeting PI3K/Akt [31]. Furthermore, Licochalcone A, extracted from the root of *Glycyrrhiza inflata* inhibits cell proliferation, migration, and invasion by suppressing the PI3K/Akt signaling pathway in oral squamous cell carcinoma [32]. Previous studies demonstrated PI3K/Akt pathway activation induces E-cadherin expression and inhibits vimentin expression to suppress cancer cell proliferation and migration [33, 34]. Our study observed that Tan IIA downregulated the expression of PI3K and p-Akt by mimicking by LY294002 as a common PI3K inhibitor. Due to its association with increased cell proliferation and survival, elevated expression of PI3K-related proteins is considered a hallmark of cancer [35]. PI3K transduces extracellular signals from G protein-coupled receptors (GPCRs) to activate multiple downstream signaling proteins, including Akt (also known as protein kinase B). Akt phosphorylates various downstream effectors, including mTOR and glycogen synthase kinase 3(GSK-3), contributing to cancer cell growth, proliferation, and survival [30]. Previous studies had confirmed that Tan IIA affects malignant cell growth by inhibiting the PI3K/Akt/mTOR pathway [17, 36, 37]. Although current study did not detect the expression of mTOR, we speculate that the mechanism by which Tan IIA inhibits SCLC metastasis is associated with mTOR suppression. Therefore, this hypothesis remains to be tested.

Using an in vivo xenografted tumor model in mice, we showed that the tumor volume and weight were reduced after Tan IIA administration without cytotoxic effects based on the results of serum biochemical analysis and body weight measurements. The number of mice in each group was limited, however, we observed that the Tan IIA dosage showed low toxicity in mice consistented with in vitro results. Also, in accordance with the in vitro results, we found that the expression of E-cadherin was increased by Tan IIA, while the expression of PI3K and p-Akt was suppressed in vivo.

However, our experimental design had some shortcomings. First, the anti-tumor effect of Tan IIA could not be identified while blocking the PI3K/Akt pathway in the tumor xenograft animal model. Second, although Tan IIA could inhibit the PI3K/Akt signaling pathway, its specific target of Tan IIA of suppressing the expression of PI3K and p-Akt expression still needs further investigation. Third, RNA-sequencing revealed that Tan IIA treatment downregulated certain DEGs. However, we did not explore the impact of specific genes on anti-tumor, which will be investigated in our future study.

## Conclusion

In summary, our study showed that Tan IIA suppresses the proliferation and metastasis of SCLC by inhibiting the PI3K/Akt signaling pathway, thus highlighting Tan IIA as a potential therapeutic drug for SCLC.

### Electronic supplementary material

Below is the link to the electronic supplementary material.

Supplementary Material 1

Supplementary Material 2

Supplementary Material 3

Supplementary Material 4

Supplementary Material 5

## Data Availability

The datasets generated and analyzed during the current study are available from the corresponding author on reasonable request.

## Abbreviations

DEGs: Differentially expressed genes
DMEM: Dulbecco’s modified Eagle’s medium
DMSO: Dimethyl sulfoxide
EMT: Epithelial-to-mesenchymal transition
FBS: Fetal bovine serum
HBE: Human normal bronchial epithelial cell line
HRP: Horseradish peroxidase
KEGG: Kyoto Encyclopedia of Genes and Genomes
NSCLC: Non-small cell lung cancer
PI: Propidium iodide
PVDF: Polyvinylidene fluoride
RIPA: Radioimmunoprecipitation
RPMI: Roswell Park Memorial Institute
SDS-PAGE: Sodium dodecyl sulfate polyacrylamide gel electrophoresis
SCLC: Small cell lung cancer
Tan IIA: Tanshinone IIA
TPM: Transcripts per million reads
