# Supplementary material for: Tanshinone IIA inhibits proliferation and migration by downregulation of the PI3K/Akt pathway in small cell lung cancer cells
Source: BMC Complement Med Ther. 2024 Jan 31;24:68. doi: 10.1186/s12906-024-04363-y (PMC10829381; doi:10.1186/s12906-024-04363-y)
Supplement: Supplementary file 1 — Supplementary Material 1 [file 12906_2024_4363_MOESM1_ESM.pdf]

# Figure 4A

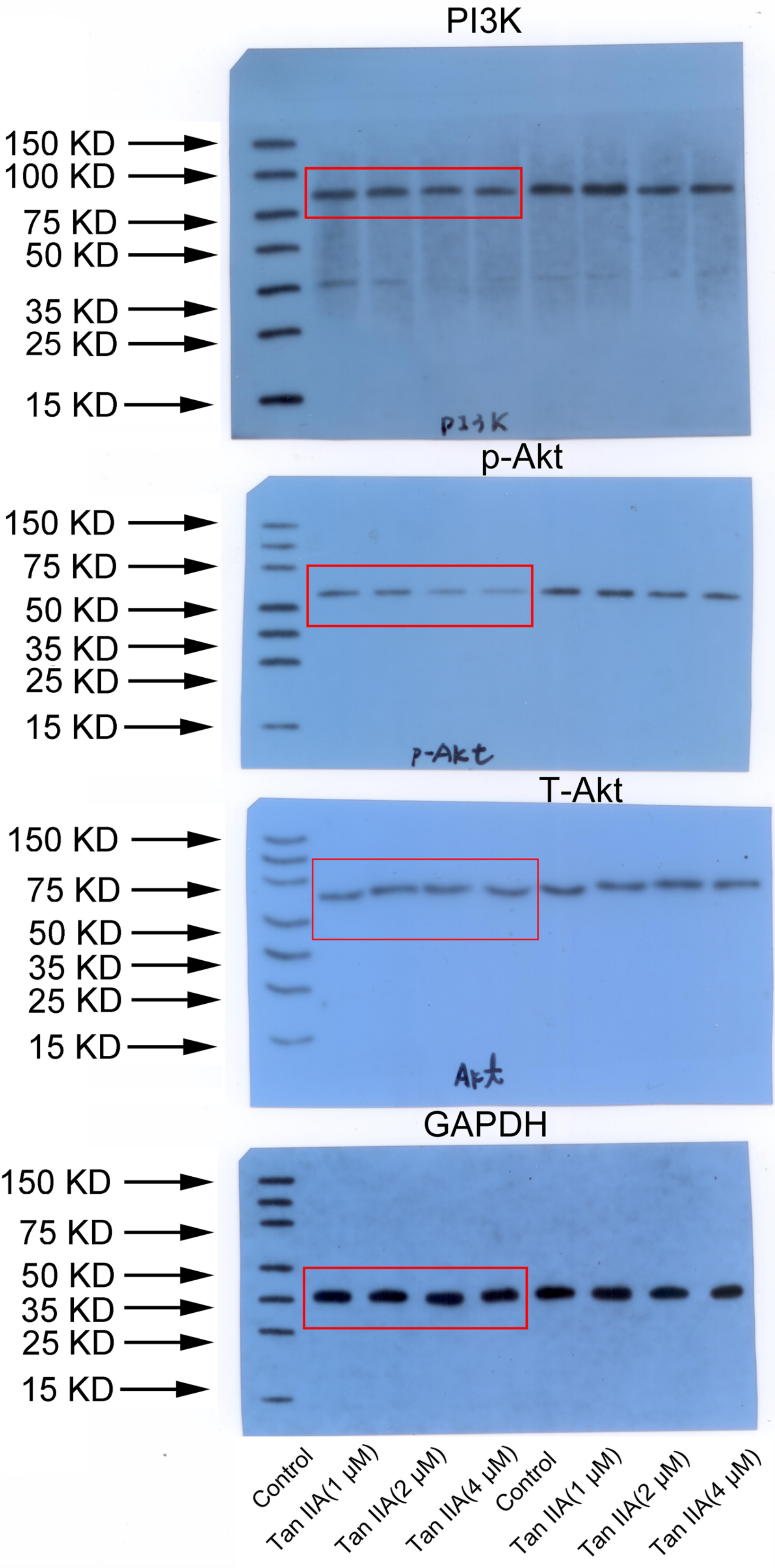

PI3K
150 KD
100 KD
75 KD
50 KD
35 KD
25 KD
15 KD
p-Akt
150 KD
75 KD
50 KD
35 KD
25 KD
15 KD
T-Akt
150 KD
75 KD
50 KD
35 KD
25 KD
15 KD
GAPDH
150 KD
75 KD
50 KD
35 KD
25 KD
15 KD
Control
Tan IIA(1 μM)
Tan IIA(2 μM)
Tan IIA(4 μM)
Control
Tan IIA(1 μM)
Tan IIA(2 μM)
Tan IIA(4 μM)
