# Supplementary figures and images for: Tanshinone IIA inhibits proliferation and migration by downregulation of the PI3K/Akt pathway in small cell lung cancer cells
Source: BMC Complement Med Ther. 2024 Jan 31;24:68. doi: 10.1186/s12906-024-04363-y (PMC10829381; doi:10.1186/s12906-024-04363-y)

Figure 4B

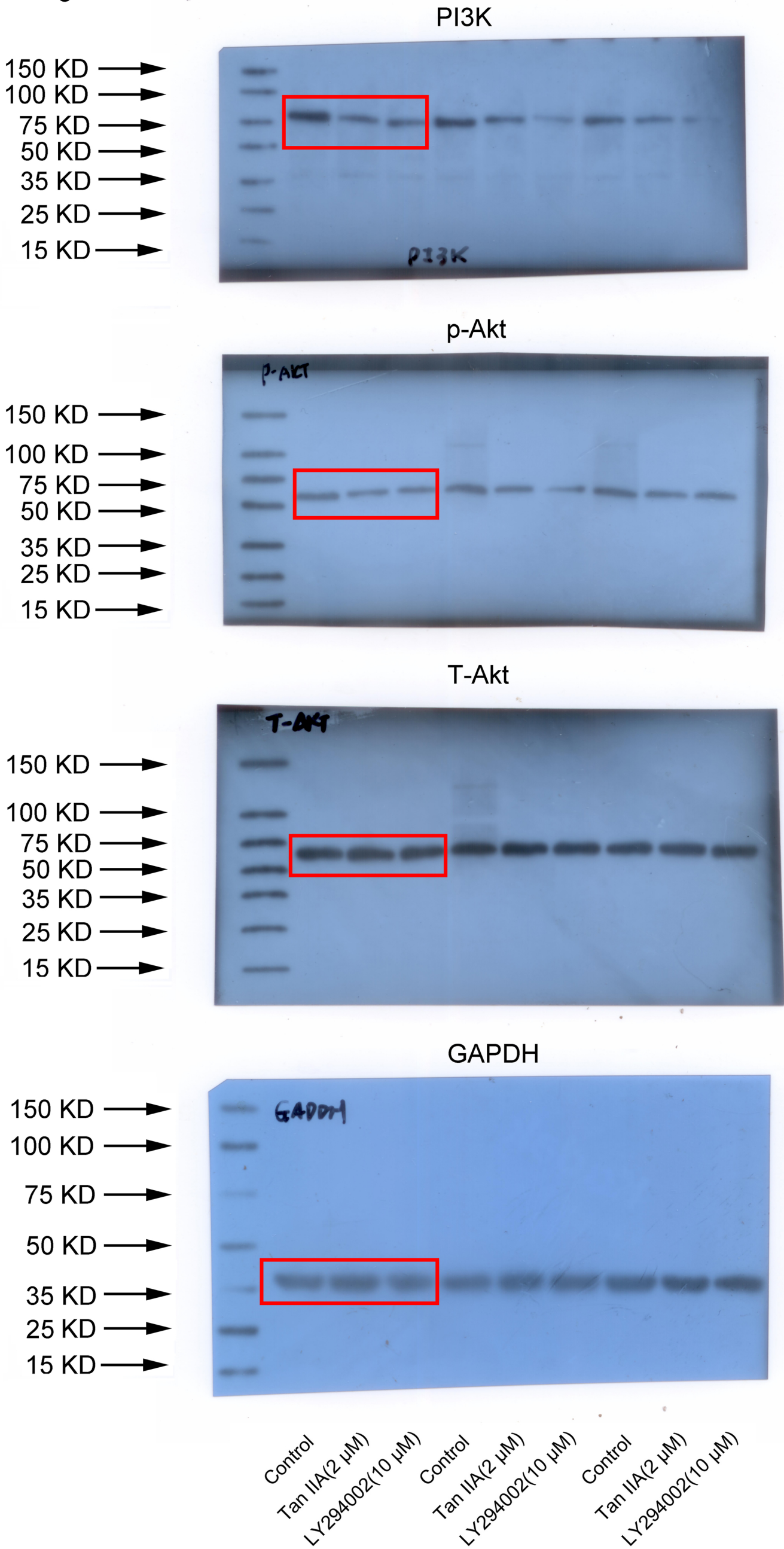

Supplement: Supplementary file 2 — Supplementary Material 2 [file 12906_2024_4363_MOESM2_ESM.pdf]

Figure 4C

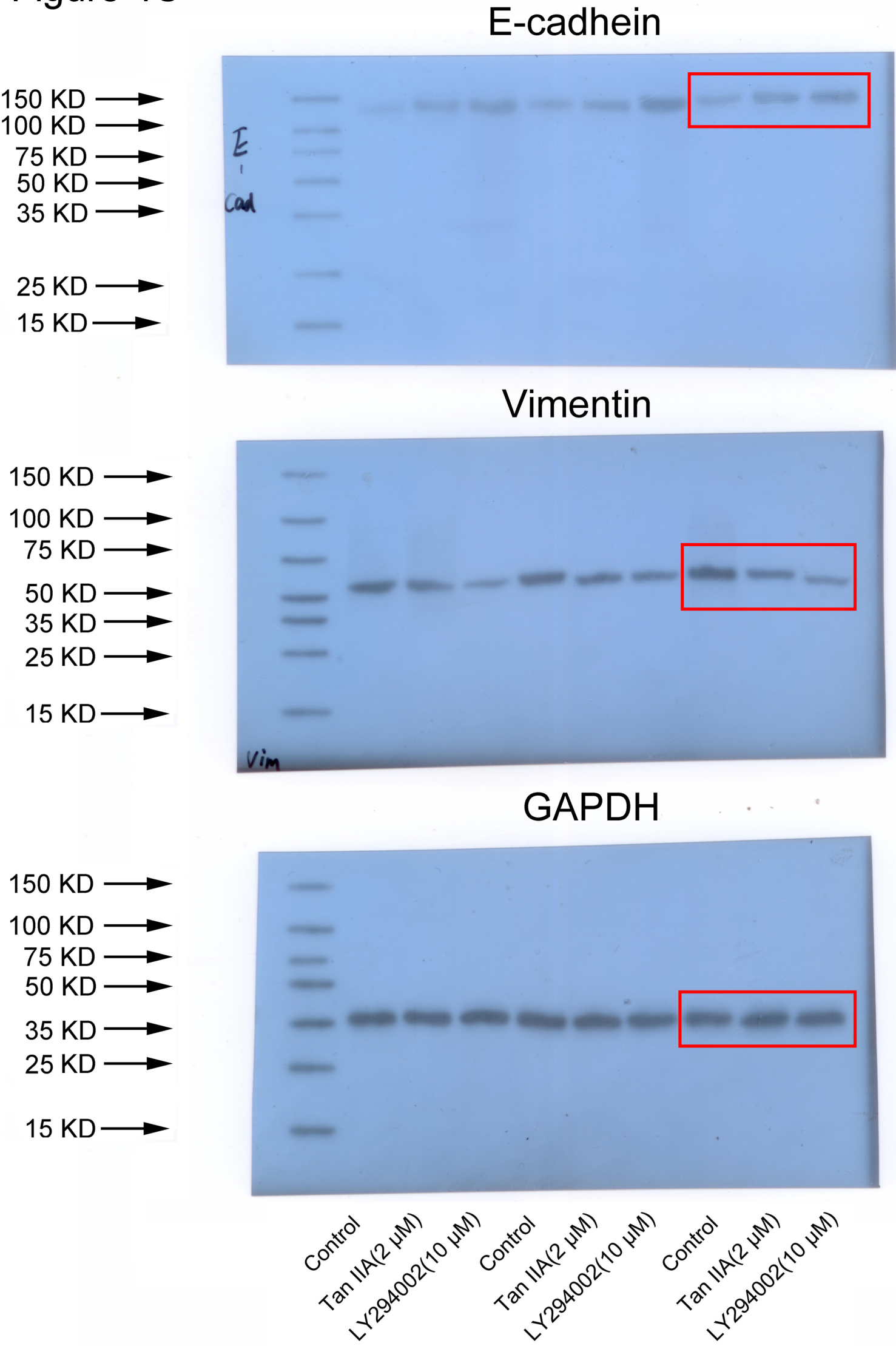

Supplement: Supplementary file 3 — Supplementary Material 3 [file 12906_2024_4363_MOESM3_ESM.pdf]
